# Quantum Probes for Ohmic Environments at Thermal Equilibrium

**DOI:** 10.3390/e21050486

**Published:** 2019-05-12

**Authors:** Fahimeh Salari Sehdaran, Matteo Bina, Claudia Benedetti, Matteo G. A. Paris

**Affiliations:** 1 Faculty of Physics, Shahid Bahonar University of Kerman, Kerman 76169-14111, Iran; 2 Quantum Technology Lab, Dipartimento di Fisica “Aldo Pontremoli”, Università di Milano, I-20133 Milano, Italy

**Keywords:** open quantum systems, quantum probes, ohmic environments

## Abstract

It is often the case that the environment of a quantum system may be described as a bath of oscillators with an ohmic density of states. In turn, the precise characterization of these classes of environments is a crucial tool to engineer decoherence or to tailor quantum information protocols. Recently, the use of quantum probes in characterizing ohmic environments at zero-temperature has been discussed, showing that a single qubit provides precise estimation of the cutoff frequency. On the other hand, thermal noise often spoil quantum probing schemes, and for this reason we here extend the analysis to a complex system at thermal equilibrium. In particular, we discuss the interplay between thermal fluctuations and time evolution in determining the precision attainable by quantum probes. Our results show that the presence of thermal fluctuations degrades the precision for low values of the cutoff frequency, i.e., values of the order $\omega_{c}≲T$ (in natural units). For larger values of $\omega_{c}$, decoherence is mostly due to the structure of environment, rather than thermal fluctuations, such that quantum probing by a single qubit is still an effective estimation procedure.

## 1. Introduction

In the last decade, technological advances in control and manipulation of quantum systems have made quantum probes available to the characterization of a large set of physical platforms. In turn, a radically new approach to probe complex quantum systems emerged, and it is based on the quantification and optimization of the information that can be extracted by an immersed quantum probe, as opposed to a classical one [1,2,3,4,5,6,7,8,9]. Quantum probes offer two main advantages: on one hand, they often provide enhanced precision, due to the inherent sensitivity of quantum system to environment-induced decoherence. On the other hand, they provide non-invasive techniques in order to estimate parameters of interest, without perturbing the system under investigation too much.

In this paper, we address the use of the simplest quantum probe, a single qubit, as it already embodies all the desired properties of an effective probe: it is small, only weakly invasive, and it can be easily manipulated and controlled [10,11,12]. Our aim is to characterize the spectral properties of a bath of oscillators, which itself provides a quite general model, suitable to describe several complex systems of interest for quantum information science and reservoir engineering [13,14,15,16,17,18]. In particular, we focus on the cutoff frequency $\omega_{c}$ of the environment, which is linked to the environment correlation time and, in turn, to the available coherence time for communication and computation. Indeed, a precise characterization of the spectral density is a crucial step to the engineering of reservoirs, tailored to specific tasks. Recently, the effective use of a single qubit quantum probe to characterize ohmic environments at zero temperature has been analyzed and discussed [3]. On the other hand, thermal fluctuations often spoil the effectiveness of quantum metrological protocols, the most dramatic case being represented by quantum interferometry, where an infinitesimal amount of noise is enough to kill Heisenberg scaling and reinstates the shot noise limit [19]. In turn, the effect of temperature has been analyzed in different metrological contexts, for example the out-of-equilibrium regimes [20] and phase estimation in Gaussian states [21]. For these reasons, we extend here the analysis to the more realistic case of complex systems at thermal equilibrium and discuss in detail the interplay among thermal fluctuations and time evolution in making the qubit an effective probe for the cutoff frequency of its environment. In this context, a relevant feature of our probing technique is the pure dephasing nature of the interaction between the qubit and its environment. This means that, while the ohmic system has a fixed temperature, the probe has access to the full set of out-of-equilibrium states [22], while not exchanging energy with the ohmic system. As we will see, this provides room to optimize the probing strategy and to enhance sensitivity over classical (thermal) probes.

Any probing strategy requires control of the initial state of the probing system, as well as of the coupling with the probed one. Concerning the detection of the probe after interaction, we exploit results from local quantum estimation theory (QET), which provides the necessary tools to determine the most informative measurement and the most precise estimator and, in turn, to optimize the extraction of information from the quantum probe [23]. Indeed, QET has been effectively employed in different contexts [24,25,26,27,28,29,30,31,32,33,34,35,36,37,38,39,40,41,42,43,44], in order to individuate the most convenient detection scheme and to evaluate the ultimate quantum bounds to precision. In this work, we address the characterization of ohmic environments at thermal equilibrium, i.e., the estimation of their cutoff frequency, assuming that the nature of the environment is known, i.e., the value of the ohmicity parameter. On the other hand, we optimize the strategy over the initial preparation of the probe qubit, the interaction time, and the detection scheme at the output. In particular, we pay attention to the overall estimability of the cutoff frequency, as measured by the quantum signal-to-noise ratio, in different temperature regimes. As we will see, the presence of thermal fluctuations degrades the estimation precision. On the other hand, the negative effects of temperature are relevant only for small values of the cutoff frequency, i.e., values of the order $\omega_{c}≲T$ (in natural units). For larger values of the cutoff frequency, the decoherence of the probe is mostly due to the structure of the environment, rather than thermal fluctuations, so the overall estimation procedure is still very effective, with performances very close to the zero temperature case.

The paper is structured as follows. In Section 2, we describe the interaction model, establish notation, and briefly review the ideas and the tools of QET. In Section 3, we present our results and discuss in details the interplay between thermal fluctuations and time evolution in determining the precision of quantum probes. Section 4 closes the paper with some concluding remarks.

## 2. The Model

Our quantum probe is a single qubit with energy gap $\omega_{0}$, which interacts with a bosonic reservoir at thermal equilibrium. The total Hamiltonian may be written as

(1) $$H=\frac{\omega_{0}}{2}\sigma_{3}+\sum_{k}\omega_{k}\;b_{k}^{†}\;b_{k}+\sigma_{3}\;\sum_{k}\left(g_{k}\;b_{k}^{†}+g_{k}^{*}\;b_{k}\right)\;$$

where $\omega_{k}$ is the frequency of the *k*-th reservoir mode, and we use natural units with $\hbar =k_{B}=1$. The Pauli matrix $\sigma_{3}$ acts on the qubit, and $\left[b_{k},b_{k}^{†}\right]=\delta_{k\;k^{{}^{\prime }}}$ describes the modes of the bath. The $g_{k}$’s are coupling constants, describing the interaction of each mode with the qubit. Their distribution is usually described in terms of the so-called spectral density of the bath, which is defined as $J\left(\omega \right)=\sum_{k}\;{\left|g_{k}\right|}^{2}\;\delta \left(\omega_{k}-\omega \right)$. The spectral density depends on the the specific features of the physical system and may often be engineered in order to enable control of quantum decoherence [2]. The model described by H in Equation (1) is exactly solvable, making it possible to analyze the mechanisms creating entanglement between the qubit and environment, which in turn is at the core of decoherence processes [1,2].

We are interested in probing properties of the environment by performing measurements on the qubit. To this aim, we study the reduced dynamics of the qubit assuming that the environment is at thermal equilibrium, i.e.,

$$\rho_{E}=\frac{1}{Z}\exp\left\{-\frac{1}{T}\sum_{k}\omega_{k}\;b_{k}^{†}\;b_{k}\right\}\;$$

where $Z=\mathrm{Tr}\left[\exp\{-\frac{1}{T}\sum_{k}\omega_{k}\;b_{k}^{†}\;b_{k}\}\right]$ is the partition function and *T* denotes the temperature. In particular, our goal is to probe the cutoff frequency of ohmic environments, i.e., the quantity $\omega_{c}$ appearing in spectral densities of the form

(2) $$J_{s}\left(\omega ,\omega_{c}\right)=\omega_{c}{\left(\frac{\omega }{\omega_{c}}\right)}^{s}\exp\left\{-\frac{\omega }{\omega_{c}}\right\}\;.$$

The cutoff frequency is a crucial parameter for applications in quantum information science, since it is linked to the environment correlation time and, in turn, to the available coherence time for communication and computation. The quantity *s* is a real positive number, which instead governs the behavior of the spectral density at low frequencies. Upon varying *s*, we move from the sub-ohmic (s<1), to the ohmic (s=1), and to the super-ohmic (s>1) regimes. In the following, in order to make some explicit quantitative statements, we will often refer to the paradigmatic values s=0.5,1,3 [45,46].

The initial state of the combined system, qubit and environment, is described by the density matrix

(3) $$\rho_{\mathrm{QE}}\left(0\right)=\rho_{Q}\left(0\right)⊗\rho_{E}$$

where $\rho_{E}$ is given above. The initial preparation of the qubit probe $\rho_{Q}\left(0\right)$ should be optimized in order to extract the maximum possible information on $\omega_{c}$ from measurements performed on the qubit after the interaction with the environment. This optimization has been performed in [3] for environments at zero temperature. The proof does not depend on the structure of the environment, but only on the pure dephasing map of the qubit. Since the same dynamical map is considered here, the proof holds also for thermal environments, so we consider $\rho_{Q}\left(0\right)=\left|+\rangle \langle +\right|=\frac{1}{2}\;\left(I+\sigma_{1}\right)$, where $|+\rangle =\frac{1}{\sqrt{2}}\;(|0\rangle +\left|1\rangle \right)$, {|0〉,|1〉} being the computational basis, i.e., the eigenstates of $\sigma_{3}$. We now move to the interaction picture, where the Hamiltonian and the evolution operator take on the expressions

(4) $$\begin{array}{cc} H_{I} & =\sigma_{3}\sum_{k}\left(g_{k}b_{k}^{†}e^{i\omega_{k}\tau }+g_{k}^{*}b_{k}e^{-i\omega_{k}\tau }\right) \end{array}$$

(5) $$\begin{array}{cc} U_{I}\left(\tau \right) & \propto \exp\left[\frac{1}{2}\sigma_{3}\sum_{k}\left(\alpha_{k}b_{k}^{†}-\alpha_{k}^{*}b_{k}\right)\right] \end{array}$$

where $\alpha_{k}=2g_{k}\frac{1-e^{i\omega_{k}\tau }}{\omega_{k}}$ [1]. If we assume a continuum of the environment’s modes, we can use the spectral density (2) to evaluate the evolved state of the qubit probe upon tracing out the environment $\rho_{Q}\left(\tau \right)={\mathrm{Tr}}_{E}\left[U_{I}\left(\tau \right)\;\rho_{\mathrm{QE}}\left(0\right)\;U_{I}^{†}\left(\tau \right)\right]$, which explicitly reads

(6) $$\begin{array}{cc} \rho_{Q}\left(\tau \right) & =\frac{1}{2}\left(I+e^{-\Gamma_{s}\left(\tau ,T,\omega_{c}\right)}\sigma_{1}\right)\; \end{array}$$

where

(7) $$\begin{array}{c} \Gamma_{s}\left(\tau ,T,\omega_{c}\right)=\;\;\;\int_{0}^{\infty }\;\;\;\;d\omega \;J_{s}\left(\omega ,\omega_{c}\right)\;\frac{1-\cos\omega \tau }{\omega^{2}}\coth\left(\frac{\omega }{2T}\right)\; \end{array}$$

is usually referred to as the *decoherence function*, with $\exp\{-\Gamma_{s}\left(\tau ,T,\omega_{c}\right)\}$ referred to as the decoherence factor. Notice that in Equation (7) frequencies, time and temperature are dimensionless quantities expressed in units of the probe frequency $\omega_{0}$. The decoherence function depends on the temperature *T* of the environment and on the form of the spectral density $J_{s}\left(\omega ,\omega_{c}\right)$ [1,3], i.e., on the cutoff frequency $\omega_{c}$ and the ohmicity parameter *s*. An analytic form of the integral in Equation (7) may be obtained at any temperature, time, and ohmicity parameter by expanding the hyperbolic cotangent $\coth\left(x\right)=1+2\sum_{n=1}^{\infty }e^{-2nx}$. The decoherence function may then be written as

$$\Gamma_{s}\left(\tau ,T,\omega_{c}\right)=\Gamma_{s}\left(\tau ,0,\omega_{c}\right)+2\sum_{n=1}^{\infty }{\left(\frac{T}{T+n\;\omega_{c}}\right)}^{s-1}\Gamma_{s}\left(\tau ,0,\frac{T\omega_{c}}{T+n\;\omega_{c}}\right)\;,$$

which explicitly reads

(8) $$\Gamma_{s}\left(\tau ,T,\omega_{c}\right)\;=\;\Gamma_{s}\left(\tau ,0,\omega_{c}\right)+s\left(s-1\right){\left(\frac{T}{\omega_{c}}\right)}^{s-1}\;\;\frac{\Gamma_{e}{\left[s-1\right]}^{2}}{\Gamma_{e}\left[s+1\right]}F\left(\zeta \right)$$

where $\Gamma_{e}\left[z\right]=\int_{0}^{\infty }\;dt\;t^{z-1}e^{-t}$ is the Euler Gamma function and where we introduced the function

(9) F(ζ)≡2ζ[s−1,1+Re(w)]−ζ[s−1,1+w]−ζ[s−1,1+w*]

where $w\equiv T\;\omega_{c}^{-1}+i\;T\;\tau$, $\zeta \left[p,q\right]=\sum_{k=0}^{\infty }{\left(k+q\right)}^{-p}$ is the generalized (Hurwitz) zeta function and $\Gamma_{s}\left(\tau ,0,\omega_{c}\right)$ is the decoherence function at zero temperature, i.e., [3]

(10) $$\Gamma_{s}\left(\tau ,0,\omega_{c}\right)\;=\;\Gamma_{e}\left[s\;-\;1\right]\;\left\{\;1\;-\;\frac{\cos\left[\left(s\;-\;1\right)\arctan\left(\omega_{c}\tau \right)\right]}{{\left(1+\omega_{c}^{2}\;\tau^{2}\right)}^{\frac{s-1}{2}}}\;\;\right\}\;.$$

**Figure 1:**
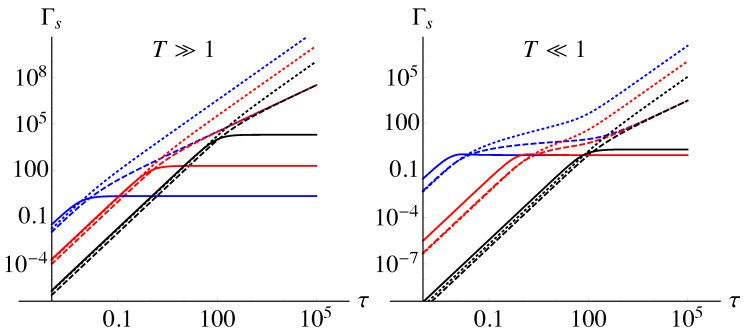
Decoherence function $\Gamma_{s}$ as a function of the dimensionless time τ for different temperatures, cutoff frequencies, and ohmicity parameters. The left panel reports $\Gamma_{s}$ in the high temperature regime (the plot is for $T=10^{2}$), whereas the right panel shows it for low temperature, $T=10^{-2}$. In both plots, black lines are for $\omega_{c}=10^{-2}$, red ones for $\omega_{c}=1$, and blue ones for $\omega_{c}=10^{2}$. Finally, solid lines denote results obtained for super-ohmic environments (s=3), dashed for ohmic (s=1), and dotted ones for sub-ohmic (s=0.5).

The behavior of the decoherence function, which from now on we denote as $\Gamma_{s}\equiv \Gamma_{s}\left(\tau ,T,\omega_{c}\right)$, as a function of the dimensionless time τ is shown in Figure 1, for different cutoff frequencies, ohmicity parameters, and two regimes of high and low temperature of the environment. As is apparent from the plots, for short times (τ≪1), the decoherence function follows a power-law scaling for any value of the other parameters. More precisely, from a first-order approximation, it scales as $\tau^{2}$:

(11) $$\Gamma_{s}\;≃\;\frac{1}{2}\;\omega_{c}^{2}\Gamma_{e}\left(s-1\right)\;\left[2{\left(\frac{T}{\omega_{c}}\right)}^{s+1}\;\;\;\;\;\zeta \left(\;s\;+\;1,\frac{T}{\omega_{c}}\right)-1\right]\tau^{2}\;.$$

The asymptotic behavior at long times, instead, is different for the three choices of the ohmicity parameters. In particular, in the super-ohmic case with s=3, the decoherence function saturates to a constant value, at any temperature *T*. This means that the stationary state of the qubit is not a fully dephased one and that the residual degree of coherence is larger for values of the parameters leading to smaller saturation values of $\Gamma_{3}$. In the other cases, sub-ohmic with s=0.5 and ohmic with s=1, the decoherence function scales, respectively, as $\Gamma_{0.5}∼\tau^{\frac{3}{2}}$ and $\Gamma_{1}∼\tau$, meaning that the stationary state of the qubit probe has been completely decohered. The long-time behavior of the decoherence function is also important from the point of view of the characterization of the type of ohmic-like environment, namely the asymptotic scaling clearly distinguishes and characterizes the ohmicity parameter of the considered structured reservoir.

### Quantum Parameter Estimation

The density matrix $\rho_{Q}\left(\tau ,\omega_{c},s,T\right)$ in Equation (6) describes the state of the qubit probe after the interaction with the environment. As such, it depends on the interaction time τ, which is basically a free parameter, on the temperature *T* and the ohmicity parameter *s*, which are fixed by the experimental conditions, and on the cutoff frequency $\omega_{c}$ of the environment, which is the parameter we would like to estimate. In the jargon of quantum estimation, it is usually referred to as a *quantum statistical model*. According to this classification, and in order to simplify the notation, in this section, we will use the following shorthands

$$\begin{array}{cc} \rho_{Q}\left(\tau ,\omega_{c},s,T\right) & ⟶\rho_{c}\;\;\frac{\partial }{\partial \omega_{c}}⟶\partial_{c}\;. \end{array}$$

Our task is to optimize the inference of $\omega_{c}$ by performing measurements on $\rho_{c}$. To this aim, we employ results from quantum estimation theory [23], which provides tools to find the best detection scheme and to evaluate the corresponding lower bounds to precision. We assume that the value of the temperature *T* and the ohmicity parameter *s* are fixed, whereas the value of interaction time is a free parameter, over which we may further optimize the precision.

Let us consider the family of quantum states $\rho_{c}$, which is labeled by the cutoff frequency $\omega_{c}$. In order to estimate $\omega_{c}$, we perform measurements on repeated preparations of the quantum probe and then process the overall sample of outcomes. The measurement *X* is any measure that can be performed on the system. For example, it can be a polarization measurement if the system is a qubit implemented by a polarized photon. Let us denote by *X* the observable measured on the probe, and by p(x|c) the conditional distribution of its outcomes when the true value of the cutoff frequency is $\omega_{c}$. We also denote by *M* the number of repeated measurements. Once *X* is chosen and a set of outcomes $x=\{x_{1},\ldots ,x_{M}\}$ is collected, we process the data by an *estimator* ${\hat{\omega }}_{c}\equiv {\hat{\omega }}_{c}\left(x\right)$, i.e., a function from the space of datasets to the manifold of the parameter values. The *estimate* of the cutoff frequency is the average value of the estimator over data, whereas the *precision* of this estimate corresponds to the variance of the estimator i.e.

(12) $$\begin{array}{c} {\bar{\omega }}_{c}=\int \;\;dx\;p\left(x|c\right)\;{\hat{\omega }}_{c}\left(x\right)\;,\;V_{c}\equiv \mathrm{Var}\;\omega_{c}=\int \;\;dx\;p\left(x|c\right)\;{\left[{\hat{\omega }}_{c}\left(x\right)-{\bar{\omega }}_{c}\right]}^{2}\; \end{array}$$

where $p\left(x|c\right)=\Pi_{k=1}^{M}\;p\left(x_{k}|c\right)$, since the repeated measurements are independent on each other. The smaller $V_{c}$ is, the more precise the estimator is. In fact, there is a bound to the precision of any unbiased estimator (those satisfying the condition ${\bar{\omega }}_{c}\to \omega_{c}$ for M≫1), given by the Cramér-Rao (CR) inequality:

(13) $$V_{c}\geq \frac{1}{MF_{c}}\;,\;F_{c}=\int \;\;dx\;p\left(x|c\right){\left[\partial_{c}\log p\left(x|c\right)\right]}^{2}\;$$

where $F_{c}$ is the (single-measurement) Fisher information (FI). The best, i.e., more precise, measurement to infer the value of $\omega_{c}$ is the measurement maximizing the FI, where the maximization should be performed over all possible observables of the probe. To this aim, one introduces the symmetric logarithmic derivative $L_{\omega_{c}}\equiv L_{c}$ (SLD), as the operator which satisfies the relation

(14) $$L_{c}\;\rho_{c}+\rho_{c}\;L_{c}=2\partial_{c}\rho_{c}\;.$$

The quantum CR theorem states that the optimal quantum measurements are those corresponding to the spectral measure of the SLD, and consequently $F_{c}\leq H_{c}=\mathrm{Tr}\left[\rho_{c}\;L_{c}^{2}\right]$, where $H_{c}$ is usually referred to as the quantum Fisher information (QFI). The quantum CR inequality then follows

(15) $$V_{c}\geq \frac{1}{MH_{c}}\;,$$

and it represents the ultimate bound to precision, taking into account both the intrinsic (quantum) and extrinsic (statistical) source of fluctuations for the estimator. Starting from the diagonal form of the quantum statistical model $\rho_{c}=\sum_{n}\rho_{n}\;|\phi_{n}\rangle \langle \phi_{n}|$, where both the eigenvalues and the eigenvectors do, in general, depend on the parameter of interest, we arrive at a convenient form of the QFI

(16) $$H_{c}=\sum_{n}\frac{{\left(\partial_{c}\rho_{n}\right)}^{2}}{\rho_{n}}+2\sum_{n\neq m}\frac{{\left(\rho_{n}-\rho_{m}\right)}^{2}}{\rho_{n}+\rho_{m}}\;|\langle \phi_{m}|\partial_{c}\;\phi_{n}\rangle {|}^{2}\;$$

where, for our qubit case, n,m=1,2. The first term in Equation (16) is the FI of the distribution of the eigenvalues $\rho_{n}$, whereas the second term is a positive definite, genuinely quantum, contribution, explicitly quantifying the potential quantum enhancement of precision. Any measurement *X* on the system is associated to its FI, and different measurements lead to different degrees of precisions through the CR bound. However, when a measurement is found, such that the condition $F_{c}=H_{c}$ is satisfied, the measurement is said to be *optimal*. If the equality in Equation (15) is satisfied, the corresponding estimator is said to be *efficient*. A global measure of the estimability of a parameter, weighting the variance with the value of the parameter, is given by the signal-to-noise ratio $R_{c}=\omega_{c}^{2}/V_{c}$. The quantum CR bound may then be rewritten in terms of $R_{c}$, as follows:

(17) $$\begin{array}{c} R_{c}\leq Q_{c}=\omega_{c}^{2}H_{c}\; \end{array}$$

where $Q_{c}$ is referred to as the quantum signal-to-noise ratio (QSNR), and itself represents the ultimate quantum bound to the estimability of a parameter [9,23]. The larger the QSNR is, the (potentially) more effective the estimation scheme is [3]. Here “potentially” refers to the fact that a large value of the QSNR means a large QFI, which in turn tells us about the maximum precision that can be achieved. However, it does not say anything about the best estimator that must be employed in order to process the output data and to infer the value of the parameter. A large $Q_{c}$ is a necessary step in order to precisely estimate the parameter.

Finally, we notice that $\omega_{c}$ takes a value on a subset of the real axis, and this means that, even if the optimal measurement does depend on the value to be estimated, the ultimate precision dictated by the quantum Cramer–Rao bound may be achieved by a two-stage adaptive scheme [47].

## 3. Quantum Probes for Ohmic Environments at Thermal Equlibrium

In this section, using results of Section 2, we discuss the performances of a qubit probe in estimating the cutoff frequency of ohmic environments at thermal equilibrium. Our starting point is the state of the probe after the interaction with the environment, which provides the quantum statistical model $\rho_{c}$. We assume that the temperature *T* and the ohmicity parameter *s* are fixed by the experimental conditions, whereas the interaction time τ may be tuned in order to maximize the quantum Fisher information $H_{c}$ and, in turn, the quantum signal-to-noise ratio $Q_{c}$. To this aim, we first diagonalize $\rho_{c}$ and then use Equation (16). After some algebra, we arrive at

(18) $$\begin{array}{c} H_{c}\left(\tau \right)=\frac{{\left[\partial_{c}\Gamma_{s}\right]}^{2}}{\exp\left[2\Gamma_{s}\right]-1}\; \end{array}$$

where we have omitted the explicit dependence on *T* and $\Gamma_{s}$ is given by the explicit analytic formula (8). Starting from Equation (18), we have maximized $Q_{c}\left(\tau \right)=\omega_{c}^{2}\;H_{c}\left(\tau \right)$ over the interaction time τ at different fixed values of *T* and *s*. In particular, we have considered three specific values of s=0.5,1,3 in order to address sub-ohmic, ohmic, and super-ohmic regimes.

**Figure 2:**
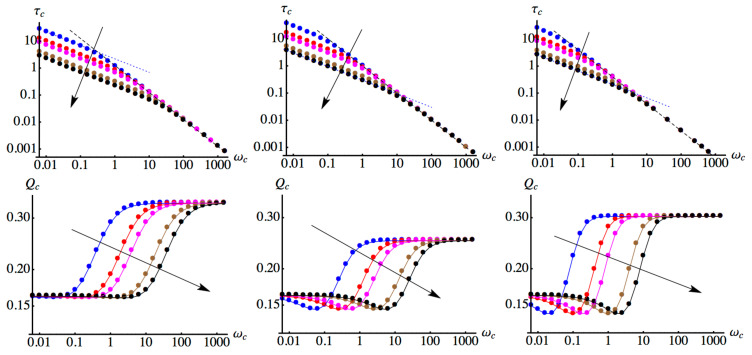
Upper plots: the optimal interaction time $\tau_{c}$ as a function of the cutoff frequency $\omega_{c}$ for different values of the temperature (from top to bottom, we have T=0.1,0.5,1.0,5.0,10.0, arrows point to increasing temperature). From left to right, the plots refer to s=0.5,1,3. Dashed lines indicate the scaling of $\tau_{c}$ with $\omega_{c}$ in the two regimes of low and high cutoff frequency. Lower plots: the optimized values of the QSNR $Q_{c}$, achieved for the interaction times of the upper plots, as a function of the cutoff frequency for different values of the temperature (from top to bottom, we have T=0.1,0.5,1.0,5.0,10.0, arrows point to increasing temperature). From left to right, the plots refer to s=0.5,1,3.

In Figure 2 we show the results of the optimization. The upper plots show the optimal interaction time $\tau_{c}$ as a function of the cutoff frequency for the three considered values of the ohmicity parameter, and for different values of temperature (T=0.1,0.5,1.0,5.0,10.0), whereas the plots in the lower panels show the corresponding optimized values of the QSNR $Q_{c}$, for the same values of *s* and *T*. In all plots, the arrow denotes increasing values of temperature. In the region of low cutoff frequencies, the decoherence of the probe qubit is governed by thermal fluctuation, rather than the structure of the environment. As a consequence, a larger interaction time, scaling as $\tau_{c}\propto \omega_{c}^{-1/2}$, is needed to imprint the maximal possible information about $\omega_{c}$ on the probe. The corresponding values of $Q_{c}$ are anyway smaller than those achievable in the zero temperature case, which corresponds to the upper saturation level for $\omega_{c}\gg T$. Upon increasing the cutoff frequency, the zero temperature scaling of the optimal time, $\tau_{c}\propto \omega_{c}^{-1}$ is recovered, as well as the values of the optimized QSNR. Combining numerical results with Equation (8) we see that for $\omega_{c}>T$ the optimal time scales as follows: $\tau_{c}≃\frac{5}{4}\;\omega_{c}^{-1}$ for s=0.5, $\tau_{c}≃\omega_{c}^{-1}$ for s=1, and $\tau_{c}≃\frac{2}{5}\;\omega_{c}^{-1}$ for s=3, independently on the temperature itself.

The transition from the regime of decoherence induced by temperature to the regime of decoherence governed by the structure of the environment may be traced back to the behavior of the decoherence function $\Gamma_{s}$, and takes place for cutoff frequencies of the order $\omega_{c}≃T$. Remarkably, as $\omega_{c}$ exceeds this threshold value, the value of the QSNR $Q_{c}$ quickly increases and reaches the zero temperature level independently on the temperature of the environment. We notice that, even in the region of low cutoff frequencies where thermal fluctuations degrade performances (the QSNR is reduced by a factor about 2/3), qubit probes are still providing information about their environment.

## 4. Conclusions

In this paper, we have addressed estimation of the cutoff frequency of a complex ohmic environment at thermal equilibrium. Our approach is based on the use of a quantum probe, i.e., a simple quantum system that undergoes decoherence due to its interaction with the environment. In particular, we have focused on the use of a single qubit subject to environment-induced dephasing and have evaluated the optimal interaction time between the probe and the environment that is needed to imprint the maximum information about the cutoff frequency onto the qubit. In addition, we have discussed the interplay between thermal fluctuations and time evolution in determining the precision of quantum probes.

Our results show that the presence of thermal fluctuations degrades the precision for low values of the cutoff frequency, whereas for larger values a single qubit is still providing nearly optimal performances, i.e., a precision close to the zero temperature case. This behavior may be explained in terms of the mechanisms responsible for the decoherence of the qubit. In the region of low cutoff frequencies, the decoherence of the probe is governed by thermal fluctuations, rather than the structure of the environment. As a consequence, a larger interaction time, scaling as $\tau_{c}\propto \omega_{c}^{-1/2}$, is needed to imprint the maximal possible information about $\omega_{c}$ onto the probe, and the corresponding values of the QSNR are smaller than those achievable in the zero temperature case. On the other hand, upon increasing the cutoff frequency, thermal fluctuations are no longer the main cause of decoherence, and the zero temperature scaling of the optimal interaction time, $\tau_{c}\propto \omega_{c}^{-1}$ is recovered, as well as the values of the optimized QSNR. Our results pave the way for possible applications to realistic room temperature systems, as well as for the estimation of more than a single parameter in system-environment couplings with general spectra.
